# Implications of the causality principle for ultra chiral metamaterials

**DOI:** 10.1038/srep09273

**Published:** 2015-03-19

**Authors:** Maxim V. Gorkunov, Vladimir E. Dmitrienko, Alexander A. Ezhov, Vladimir V. Artemov, Oleg Y. Rogov

**Affiliations:** 1 A. V. Shubnikov Institute of Crystallography, Russian Academy of Sciences, 119333 Moscow, Russia; 2 M. V. Lomonosov Moscow State University, 119991 Moscow, Russia; 3 A. V. Topchiev Institute of Petrochemical Synthesis, Russian Academy of Science, 119991 Moscow, Russia

## Abstract

Chiral metamaterials – artificial subwavelength structures with broken mirror symmetry – demonstrate outstanding degree of optical chirality that exhibits sophisticated spectral behavior and can eventually reach extreme values. Based on the fundamental causality principle we show how one can unambiguously relate the metamaterial circular dichroism and optical activity by the generalized Kramers-Kronig relations. Contrary to the conventional relations, the generalized ones provide a unique opportunity of extracting information on material-dependent zeroes of transmission coefficient in the upper half plane of complex frequency. We illustrate the merit of the formulated relations by applying them to the observed ultra chiral optical transmission spectra of subwavelength arrays of chiral holes in silver films. Apart from the possibility of precise verification of experimental data, the relations enable resolving complex eigenfrequencies of metamaterial intrinsic modes and resonances.

The absence of mirror symmetry being a common attribute of numerous natural objects and materials, especially those of biological origin, typically gives rise to very moderate optically observable consequences. Artificial chiral electromagnetic materials provide high and even sometimes extreme degrees of optical activity (OA) and circular dichroism (CD)^1^,^2^,^3^,^4^,^5^,^6^,^7^,^8^ that may also appear via spontaneous symmetry breaking^9^ and can possess additional functional properties such as strong nonlinearity^10^ and tunability by irradiation^11^. Being unattainable with natural materials, these features are highly advantageous for the potential applications that range from electromagnetic signal manipulation^12^ to nanoscale chirality diagnostics^13^.

For an artificial structure to acquire a substantial electromagnetic chirality, it has to possess a pronounced structural chirality with possibly high intrinsic electromagnetic contrast, i.e., it has to include the constituents with sufficiently different electromagnetic response. The structure period has to be subwavelength in order to provide the effective homogeneity of the material. Accordingly, chiral metamaterials – subwavelength metal-dielectric structures and arrays with broken mirror symmetry – have proved to be a very fruitful concept^14^,^15^.

While the fabrication of illustrative metamaterial samples operational in the radio and microwave ranges does not require sophisticated techniques, creating the structures with micron and submicron periodicity for the infrared and visible ranges still remains challenging. Different types of such chiral metamaterials fabricated by various techniques have been reported: multi layered structures operational at the wavelengths of a few microns^2^ and in the visible^3^, nanoscale dielectric helical templates decorated with plasmonic nanoparticles^4^, metallic helices^5^, precisely elevated starfish-shaped metal particles^6^ and arrays of chiral holes with extreme CD and OA in the visible^8^.

Independently of the scale and type of chiral medium, the CD and OA are always the key characteristics being the observables in the chirality diagnostics and defining the main functional properties in prospective applications. It has been recognized for decades that the general principle of causality in the form of appropriate Kramers-Kronig (KK) integral relations for the difference of complex refractive indices for the left and right circularly polarized waves can provide a valuable opportunity to relate CD and OA of natural materials with molecular-scaled inner structure and weak optical chirality (see e.g. Chapter 21 in Ref. 16 and refs. therein). In artificial materials, however, the much larger inner scales prevent from introducing effective macroscopic parameters. Remarkably, the reported spectral behavior of CD and OA is often nontrivial and seemingly contradicts to the conventional rules of KK-relations. According to them, a resonant peak of one characteristic should be accompanied by an antiresonant kink of its counterpart. While in some cases this holds true (see e.g. Ref. 2), in many others the situation is different and a broadband OA may appear with negligibly small CD^7^ or both OA and CD can peak together up to their extreme values at very close wavelengths^8^.

In this paper we show that the causality allows to introduce an appropriate form of the KK integral relations for the OA and CD. Being strictly correct from the mathematical point of view and based solely on the fundamental principle of causality, the relations can be widely used as a solid reference point. In addition, the correct form of the relations includes the so-called Blaschke terms that are determined by the inner resonances and modes of the chiral material. This provides a unique opportunity of extracting valuable quantitative information on important intrinsic material features by means of conventional spectropolarimetry. Using as example the spectropolarimetry data for the arrays of nanosize chiral holes in metal films we demonstrate the latter possibility and obtain explicitly the complex eigenfrequencies of the chirally split leaky waves supported by the arrays.

## Generalized Kramers-Kronig relations

Mathematically, the KK-relations connect the real and imaginary parts of a function of complex variable that is known to be analytical in the upper half-plane of the variable and approaches zero as the variable tends to infinity. In physics, a direct and simple deduction to the causality principle allows applying the KK-relations to calculate the frequency dependence of the real parts from the known imaginary parts (or vice versa) of refractive index, permittivity or susceptibility^17^. However, these well-known forms of the KK integrals are not universal and are to be replaced by more general relations if the analytical properties of the response functions are more complicated. In particular, it is often useful to consider the logarithm of the reflection or transmission amplitudes as response functions. If the amplitudes turn to zero (staying analytical) at certain complex frequencies *ω_i_* in the upper-half complex frequency plane, the KK-relations for the logarithms are to be modified accordingly by introducing the so-called Blaschke term known from the theory of analytical functions. Appearance of such situations in various physical problems was first recognized by van Kampen^18^ and then analyzed in detail by Toll^19^. The Blaschke term changes drastically the phase of the reflection and transmission coefficients hence being of key importance in the phase retrieval problems^20^,^21^,^22^.

For the problem of light transmission through metamaterial layers, the complex transmission amplitude represents a response function which Fourier transform *t*(*ω*) = |*t*(*ω*)| exp[*i*Ψ(*ω*)] is analytical in the upper-half plane of the complex frequency *ω*. However, the inappropriate high frequency limit of the electromagnetic transmittance, |*t*(*ω*)| → 1 for |*ω*| → ∞, does not allow to employ *t*(*ω*) directly in the KK-relations that require asymptotic vanishing of the response at |*ω*| → ∞. Instead, one can consider the logarithm of the transmission amplitude ln[*t*(*ω*)] = ln |*t*(*ω*)| + *i*Ψ(*ω*). Then if ln |*t*(*ω*)| is analytical everywhere in the upper half-plane of complex *ω*, the corresponding KK-relations read:
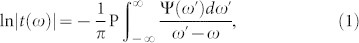

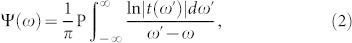
where P stands for the principal value of the integrals.

If *t*(*ω*) has one or more zero points in the upper-half plane of the complex frequency, one can consider an auxiliary function 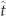 such that 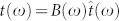, where the Blaschke multiplier *B*(*ω*) contains explicitly all *n* ≥ 1 zeros *ω_i_* of *t*(*ω*) (multiple roots considered as different roots):
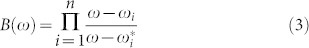
where the star means complex conjugate. Obviously |*B*(*ω*)| = 1 for real *ω* and the multiplier changes only the phase of the transmission amplitude. The analytical function 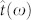 has no zeros and for its logarithm one can write the KK-relations. As a result, the KK-relations (1) and (2) for the physically meaningful |*t*| and Ψ are to be adjusted by the substitution
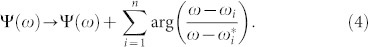


For a chiral system, one applies the above routine to the transmission amplitudes *t_R_* and *t_L_* of the right and left circularly polarized radiation respectively. Defining the observable CD (*D*) and OA (Φ) conventionally as
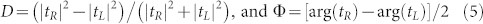
and using the identity ln[(1 + *D*)/(1 − *D*)] = 2 ln |*t_R_*| − 2 ln |*t_L_*| one can write the appropriate KK-relations as:
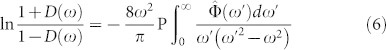

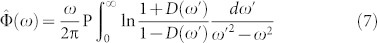
where the angle 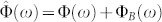 entering the KK-relations includes the Blaschke phase
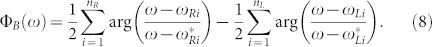
Here the summations over the zeros *ω_Ri_* and *ω_Li_* of the amplitudes *t_R_* and *t_L_* correspondingly is performed.

## Application to ultra chiral metamaterials

**Figure 1:**
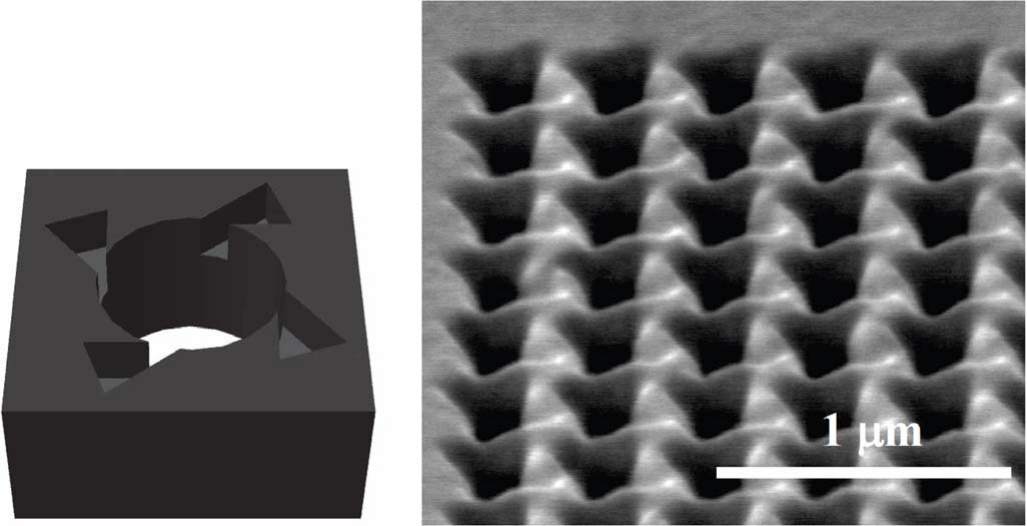
3D-model of a chiral hole as implemented into the focused ion beam milling digital template (left) and SEM image of the fabricated array A1 tilted by 52° (right).

To illustrate the application of these very general relations, we consider the experimental data on the ultra chiral metamaterial comprised of subwavelength 4-fold arrays of chiral holes in freely suspended silver foils. Technically, the experimental methods of fabrication and optical characterization used have been very similar to those reported recently in Ref. 8. Two sample arrays discussed below are of the same type, have equal lateral dimensions and were milled using FEI Helios Nanolab 650 microscope in the foils of different thickness: 270 nm (array A1) and 380 nm (array A2). Being fabricated with single-pass focused ion beam (FIB) milling according to the digital template shown schematically in Fig. 1 on the left, the arrays possess the in-plane fourth order rotational symmetry. A fragment of the fabricated array A1 is shown in Fig. 1 on the right where the difference from the template due to ion beam defocusing and diversion can be seen as well. The periods of both array square lattices were set to 375 nm to avoid diffraction in the visible, and the inner hole diameter was 187 nm. The symmetry breaking responsible for the structural chirality (the absence of mirror planes) was granted by the offset of the triangles patterned on one array interface.

**Figure 2:**
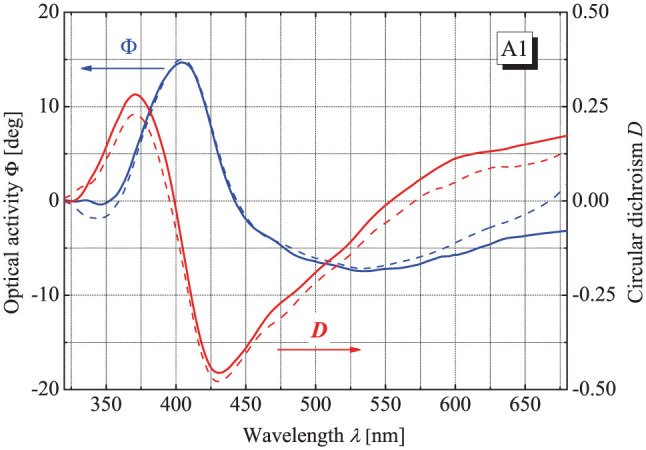
Measured CD and OA of the array A1 (solid lines) compared to CD and OA calculated according to Eqs. (6) and (7) without Blaschke terms (dashed).

**Figure 3:**
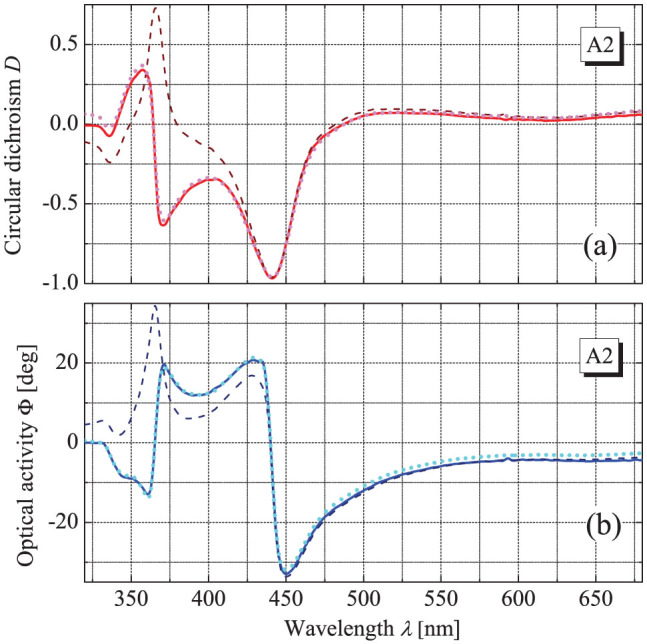
Measured CD (a) and OA (b) of the array A2 (solid lines) compared to CD and OA calculated according to Eqs. (6) and (7) without (dashed) and with (dotted) Blaschke terms being taken into account.

The microspectropolarimetry of light transmitted through the samples was carried out with a spectroscopic Uvisel 2 (Horiba Jobin-Yvon) ellipsometer as described in Ref. 8. The CD and OA data obtained for the samples A1 and A2 are shown by solid lines in Figs. 2 and 3 respectively. The optical chirality of both arrays is notably strong as the OA reaches several tens of degrees and the CD peaks down to the value of −0.5 in the thinner array A1 and almost reaches the extreme value of −1 in the thicker array A2. Remarkably, although the complex spectral behavior of the strong optical chirality seems to have much in common with the recently reported extreme optical chirality of 4-start screw thread chiral holes^8^, there exists a qualitative difference seen vividly in Fig. 2, where a peak of OA is accompanied by an antiresonance of CD. This situation is inverse compared to the data for the threaded holes^8^ and also appears to be rather unusual in general as in natural chiral materials narrow peaks of handedness-selective absorption give rise to antiresonant OA.

Next, we have calculated the integrals in Eqs. (6) and (7) numerically for the experimental data. The results of this integration obtained without accounting for possible Blaschke phase are presented as dashed lines in Figs. 2 and 3 and demonstrate clearly the real complexity of the application of casuality principle to artificial chirality. The OA and CD spectra for the thinner A1 array satisfy the simple KK-relations nicely, as the calculated and measured values practically coincide in the broad visible range (small deviations closer to the borders are related to the finite spectral range of experimental data). At the same time, the data obtained for the thicker array A2 show a dramatic discrepancy. In fact, even without doing calculations one can see that the behavior of CD and OA for the array A2 is counterintuitive: both quantities experience a pronounced antiresonance around the 370 nm wavelength. According to the conventional KK-relations, an antiresonance of one quantity has to be accompanied by a resonance of its counterpart and the dashed lines in Fig. 3 behave exactly in this manner being in total disagreement with the experiment.

**Figure 4:**
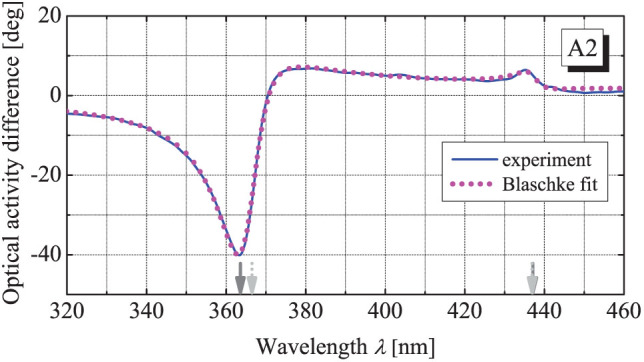
Difference of the measured OA 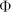 and 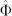 calculated from the measured CD according to Eq. (7) for the array A2 (solid) and its fit by the analytical Blaschke phase (9) (dotted) with the parameters *λ_R_*_1_ = 363.6 − 7.3*i* nm, *λ_L_*_1_ = 366.6 − 4.0*i* nm, *λ_R_*_2_ = 437.1 − 3.2*i* nm, *λ_L_*_2_ = 436.9 − 3.3*i* nm. The solid and dotted arrows indicate the real parts of *λ_Ri_* and *λ_Li_* respectively.

To clarify this issue we consider the difference between the observed Φ and 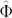 calculated from the measured CD according to Eq. (7). As shown in Fig. 4, this residue has a very particular form being obviously a sum of narrow peaks around specific wavelengths. Notably, it can be fitted precisely with the Blashke phase (8) that as a function of wavelength reads
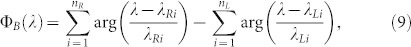
where *λ_R_*_,*Li*_ = 2*πc*/*ω_R_*_,*Li*_. As seen in Fig. 4, the Blaschke phase with the contributions from two pairs of the transmission zero points on the complex plane appears to be sufficient for the fitting. Implementing thus resolved zero points into the generalized KK-relations (6–8) allows calculating the OA from the CD and vice-versa that nicely coincide with the experiment (see dotted lines in Fig. 3).

## Discussion

As we have shown, accounting for the Blaschke phase can be critically important for the application of the KK relations to strongly chiral metamaterials. At the same time, in certain cases (as for the array A1) the absence of the zero transmission frequencies allows using a simpler version of the relations. Since the two arrays of chiral holes discussed above are very similar apart from their different thicknesses, there can hardly exist a general recipe for predicting the existence and number of such frequencies beforehand. The problem of how particularly those zeros appear as one gradually varies experimental parameters (e. g. the array thickness) is worth a separate consideration and we leave it beyond the scope of this paper. It seems quite reasonable though that thinner structures should lack certain zero transmission frequencies that thicker ones possess. One can be sure that as the thickness tends to zero, the transmission increases and its zeros disappear.

The possibility to obtain quantitative information on specific complex eigenfrequencies (wavelengths) of a metal hole array is, in fact, quite unique and valuable. Generally, the optics of metal hole and slit arrays is determined by the coexistence and interplay of different resonant phenomena such as surface plasmons (both localized and traveling)^24^, Fabry-Perot guided mode resonances^25^, Rayleigh-Wood diffraction anomalies, etc. Notably, while some absorptive resonances give rise to usual narrow peaks of extinction and deeps of transmission (see, e.g. Ref. 26), the others related to coupling between incident plane waves and traveling leaky plasmons yield asymmetric Fano-type dispersion of the transmission coefficient with distinct peaks and deeps at close wavelengths^24^.

The above analysis of the experimental data yields rather precise values of the complex zero transmission frequencies providing though not much insight into their origin. The strongest contribution to the Blaschke phase arises from the first pair of zero points, *λ_R_*_1_ and *λ_L_*_1_, which real parts are very close to the array period (375 nm ± 5% according to the SEM images). Since decades it has been recognized that special points of that kind are to be responsible for the Wood anomaly adjacent to the diffraction Rayleigh anomaly and determined by the coupling of incident light to leaky surface waves supported by arrays and gratings^23^. Eventually we have managed to measure the exact location of such points. Moreover, we have revealed also that the structural chirality causes a noticeable chiral splitting of the leaky modes spectrum and substantially affects their quality factor (as the mode *λ_R_*_1_ has almost a twice lower quality factor than the mode *λ_L_*_1_).

In contrast, another pair of special points resolved, *λ_R_*_2_ and *λ_L_*_2_, exhibits almost negligible chiral splitting which makes the corresponding contribution to the Blaschke phase noticeably weaker. Taking into account this much lower sensitivity of the underlying resonances to the structure chirality and reminding also that the wavelengths *λ_R_*_2_ and *λ_L_*_2_ considerably differ from the characteristic array sizes (thickness and period) we suggest that these points are related to well localized plasmonic resonances of certain fragments of periodic complexly shaped metal structure.

Generally, the formulated relations, being based solely on the causality principle are equally applicable to any artificial or natural chiral object and impose quantitative links between the CD and OA, which so far have been treated as independent observable characteristics of chiral metamaterials. To illustrate the practical value of the relations, one can consider their implications for chiral metamaterials of high (e.g. 4-fold) rotational symmetry designed for microwave range, where metals act as perfect electric conductors (PEC). It has been shown very generally that the CD may arise in such highly symmetric structures only accompanied by losses^8^,^27^ and thus a PEC structure possesses a negligible CD (as was also observed e.g. in Ref. 7). According to Eq. (7), zero CD yields zero 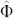 and thus the observed OA is then solely Φ = −Φ*_B_*, i.e., it exhibits the simple analytical frequency dispersion of Eq. (8) with an appropriate set of transmission zero points. Note that the numbers of zeroes for right and left circular polarizations are not necessarily equal and an unpaired zero can result in especially strong OA.

Our findings can be also directly applied to soft chiral materials such as cholesteric and blue-phase liquid crystals and polymers. It has been shown recently^28^ that in the simple planar geometry a cholesteric layer produces CD and OA that obey quite precisely the simple KK-relations of the form (6) and (7). In other geometries, however, when the Bragg vector is parallel to the entrance surface, one can expect appearance of the Blaschke term in the phase of transmitted wave. In this case, the transmission zeroes should be determined by the topological charge of the diffraction band (defined similarly to that of the x-ray diffraction bands^29^).

## Conclusion

We have shown that a strict application of the causality principle to ultra chiral metamaterials is possible in the form of generalized KK-relations. Being formulated for the main observables – CD and OA, the relations include the Blaschke terms determined by the transmission zero points on the complex frequency plane. Using as example the data for ultra chiral optical transmission of subwavelength arrays of chiral holes in silver films we have demonstrated that the KK-relations can be used for a precise verification of experimental data and, more significantly, for resolving the discrete spectrum of material-specific modes and resonances.

## Author Contributions

M.V.G. and V.E.D. developed the theoretical approach, V.V.A. and O.Y.R. designed and fabricated the samples, A.A.E. performed the optical measurements, M.V.G. processed the results, M.V.G. and V.E.D. wrote the manuscript that was discussed and improved by all authors.

## References

[b1] Rogacheva A. V., Fedotov V. A., Schwanecke A. S. & Zheludev N. I. Giant Gyrotropy due to Electromagnetic-Field Coupling in a Bilayered Chiral Structure. Phys. Rev. Lett. 97, 177401-1–177401-4 (2006). 17155505 10.1103/PhysRevLett.97.177401

[b2] Decker M., Zhao R., Soukoulis C. M., Linden S. & Wegener M. Twisted split-ring-resonator photonic metamaterial with huge optical activity. Opt. Lett. 35, 1593–1595 (2010). 20479819 10.1364/OL.35.001593

[b3] Zhao Y., Belkin M. A. & Alú A. Twisted optical metamaterials for planarized ultrathin broadband circular polarizers. Nat. Commun. 3, 870 (2012). 22643897 10.1038/ncomms1877

[b4] Singh J. H., Nair G., Ghosh A. & Ghosh A. Wafer scale fabrication of porous three-dimensional plasmonic metamaterials for the visible region: chiral and beyond. Nanoscale 5, 7224 (2013). 23832295 10.1039/c3nr02666c

[b5] Gibbs J. G., Mark A. G., Eslami S. & Fischer P. Plasmonic nanohelix metamaterials with tailorable giant circular dichroism. Appl. Phys. Lett. 103, 213101-1–213101-4 (2013).

[b6] Dietrich K. *et al.* Elevating optical activity: Efficient on-edge lithography of three-dimensional starfish metamaterial. Appl. Phys. Lett. 104, 193107-1–193107-4 (2014).

[b7] Hannam K., Powell D. A., Shadrivov I. V. & Kivshar Y. S. Broadband chiral metamaterials with large optical activity. Phys. Rev. B 89, 125105-1–125105-6 (2014).

[b8] Gorkunov M. V., Ezhov A. A., Artemov V. V., Rogov O. Y. & Yudin S. G. Extreme optical activity and circular dichroism of chiral metal hole arrays. Appl. Phys. Lett. 104, 221102-1–221102-4 (2014).

[b9] Liu M., Powell D. A., Shadrivov I. V., Lapine M. & Kivshar Y. S. Spontaneous chiral symmetry breaking in metamaterials. Nat. Commun. 5, 4441 (2014). 25033837 10.1038/ncomms5441

[b10] Ren M., Plum E., Xu J. & Zheludev N. I. Giant nonlinear optical activity in a plasmonic metamaterial. Nat. Commun. 3, 833 (2012). 22588295 10.1038/ncomms1805

[b11] Zhang S. *et al.* Photoinduced handedness switching in terahertz chiral metamolecules. Nat. Commun. 3, 942 (2012). 22781755 10.1038/ncomms1908

[b12] Turner M. D. *et al.* Miniature chiral beamsplitter based on gyroid photonic crystals. Nature Phot. 7, 801–805 (2013).

[b13] Hendry E. *et al.* Ultrasensitive detection and characterization of biomolecules using super-chiral fields. Nature Nanotech. 5, 783–787 (2010). 10.1038/nnano.2010.209 21037572

[b14] Wegener M. & Linden S. Giving light yet another new twist. Physics 2, 3 (2009).

[b15] Valev V. K., Baumberg J. J., Sibilia C. & Verbiest T. Chirality and Chiroptical Effects in Plasmonic Nanostructures: Fundamentals, Recent Progress, and Outlook. Adv. Mater. 25, 2517–2534 (2013). 23553650 10.1002/adma.201205178

[b16] King F. W. [Ch. 21 Dispersion relations for magneto-optical and natural optical activity]. Hilbert Transforms (Cambridge University Press, Cambridge, 2009).

[b17] Landau L. D. & Lifshitz E. M. Electrodynamics of Continuous Media (Pergamon, New York, 1960).

[b18] van Kampen N. G. S-Matrix and Causality Condition. I. Maxwell Field. Phys. Rev. 89, 1072–1079 (1953).

[b19] Toll J. S. Causality and the Dispersion Relation: Logical Foundations. Phys. Rev. 104, 1760–1770 (1956).

[b20] Nussenzveig H. M. Causality and Dispersion Relations (Academic, New York, 1972).

[b21] Peiponen K.-E. Dispersion theory and sumrules for the non-minimum phase problem in optical spectroscopy. Physica B 404, 2094–2096 (2009).

[b22] Peiponen K.-E. & Saarinen J. J. Generalized Kramers-Kronig relations in nonlinear optical- and THz-spectroscopy. Rep. Prog. Phys. 72, 056401-1–056401-19 (2009).

[b23] Hessel A. & Oliner A. A. A New Theory of Wood's Anomalies on Optical Gratings. Appl. Opt. 4, 1275–1297 (1965).

[b24] Genet C. & Ebbesen T. W. Light in tiny holes. Nature 445, 39–46 (2007). 17203054 10.1038/nature05350

[b25] Sturman B., Podivilov E. & Gorkunov M. Theory of extraordinary light transmission through arrays of subwavelength slits. Phys. Rev. B 77, 075106-1–075106-12 (2008).

[b26] Garcia de Abajo F. J. Colloquium: Light scattering by particle and hole arrays. Rev. Mod. Phys. 79, 1267–1290 (2007).

[b27] Kaschke J., Gansel J. K. & Wegener M. On metamaterial circular polarizers based on metal N-helices. Opt. Expr. 20, 26012–26020 (2012). 10.1364/OE.20.026012 23187416

[b28] Dolganov P. V., Ksyonz G. S., Dmitrienko V. E. & Dolganov V. K. Description of optical properties of cholesteric photonic liquid crystals based on Maxwell equations and Kramers-Kronig relations. Phys. Rev. E 87, 032506-1–032506-4 (2013).

[b29] Dmitrienko V. E. Diffraction in crystals and topology. J. Phys. I France 1, 1187–1193 (1991).

